# Beyond Seasoning—The Role of Herbs and Spices in Rheumatic Diseases

**DOI:** 10.3390/nu15122812

**Published:** 2023-06-20

**Authors:** Sofia Charneca, Ana Hernando, Patrícia Costa-Reis, Catarina Sousa Guerreiro

**Affiliations:** 1 Laboratório de Nutrição, Faculdade de Medicina, Universidade de Lisboa, Avenida Professor Egas Moniz, 1649-028 Lisboa, Portugal; sofiacharneca98@gmail.com (S.C.); ana.neves.hernando@gmail.com (A.H.); cfguerreiro@medicina.ulisboa.pt (C.S.G.); 2 Unidade de Reumatologia Pediátrica do Centro Hospitalar Universitário Lisboa Norte, Avenida Professor Egas Moniz, 1649-028 Lisboa, Portugal; 3 Instituto de Medicina Molecular João Lobo Antunes, Faculdade de Medicina, Universidade de Lisboa, Avenida Professor Egas Moniz, 1649-028 Lisboa, Portugal; 4 Clínica Universitária de Pediatria, Faculdade de Medicina, Universidade de Lisboa, Avenida Professor Egas Moniz, 1649-028 Lisboa, Portugal; 5 Instituto de Saúde Ambiental, Faculdade de Medicina, Universidade de Lisboa, Avenida Professor Egas Moniz, 1649-028 Lisboa, Portugal

**Keywords:** herbs, spices, rheumatoid arthritis, osteoarthritis, fibromyalgia, gut microbiota

## Abstract

Although we have witnessed remarkable progress in understanding the biological mechanisms that lead to the development of rheumatic diseases (RDs), remission is still not achieved in a substantial proportion of patients with the available pharmacological treatment. As a consequence, patients are increasingly looking for complementary adjuvant therapies, including dietary interventions. Herbs and spices have a long historical use, across various cultures worldwide, for both culinary and medicinal purposes. The interest in herbs and spices, beyond their seasoning properties, has dramatically grown in many immune-mediated diseases, including in RDs. Increasing evidence highlights their richness in bioactive molecules, such as sulfur-containing compounds, tannins, alkaloids, phenolic diterpenes, and vitamins, as well as their antioxidant, anti-inflammatory, antitumorigenic, and anticarcinogenic properties. Cinnamon, garlic, ginger, turmeric, and saffron are the most popular spices used in RDs and will be explored throughout this manuscript. With this paper, we intend to provide an updated review of the mechanisms whereby herbs and spices may be of interest in RDs, including through gut microbiota modulation, as well as summarize human studies investigating their effects in Rheumatoid Arthritis, Osteoarthritis, and Fibromyalgia.

## 1. Introduction

Rheumatic diseases (RDs) encompass a broad spectrum of immune-mediated disorders with a central involvement of the musculoskeletal system, causing pain, disability, and ultimately a significant decrease in health-related quality of life [1]. Although we have witnessed remarkable progress in understanding the biological mechanisms that lead to the development of RDs, the etiology of the more than 200 existing RDs remains unknown [2].

Most RDs share similar clinical and immunological manifestations, and it has become clear that genetic predisposition, environmental factors, hormonal alterations, and lifestyle factors including physical activity, diet, and stress play a pivotal role in disease onset [3]. Irrespective of the specific disease type, the affected organs are infiltrated by leukocytes, and proinflammatory cytokines are released, leading to inflammation and tissue damage [3,4]. Treatment depends on the type of RD. Pharmacological therapy is frequently used to achieve and maintain disease remission, control symptoms, and improve quality of life [5]. Over the last decades, with changes in the therapeutic approach, including the optimal use of disease-modifying anti-rheumatic drugs (DMARDs) as well as novel biological drugs, there has been an important shift to success in disease management, especially in Rheumatoid Arthritis (RA) [6]. Yet, a substantial proportion of patients with RDs are still not able to achieve remission with pharmacological drugs alone [5,7]. As a consequence, patients are increasingly looking for complementary adjuvant therapies, including dietary interventions [8].

Spices have a thousand-year history of medicinal use in India and Asia, and modern medicine has begun to explore their health-promoting potential during the last few years, with an increasing number of published articles on this subject (PUBMED: 8514 articles in 2023) [9]. The interest in herbs and spices, beyond their seasoning properties, has dramatically grown in many immune-mediated diseases due to their anti-inflammatory, antioxidant, and analgesic characteristics [10]. The potential benefits of herbs and spices have been investigated in various RDs, both in animal models and human trials. Cinnamon, garlic, ginger, turmeric, and saffron are the most popular spices used in RDs and will be further explored throughout this review. This paper aims to provide an up-to-date review of the mechanisms whereby herbs and spices may be of interest in RA, Osteoarthritis (OA), and Fibromyalgia (FM) as well as summarize human studies investigating their effects in these conditions, as they represent some of the RDs in which this topic was most explored.

## 2. The Crosstalk between Diet, Gut Microbiota, and Rheumatic Diseases

The available evidence suggests that RDs develop through a diverse range of pathophysiological pathways. The deregulation of the immune system, inflammation, and musculoskeletal damage are central phenomena in these diseases [4]. RA probably arises from a combination of environmental insults in a genetically predisposed, epigenetically modified individual, leading to a breach of immunological tolerance [11]. RA is a chronic autoimmune and inflammatory disorder marked by the presence of autoantibodies to immunoglobulin G (rheumatoid factor, RF) and citrullinated proteins (anti-citrullinated protein antibodies) [12]. 

Although human studies providing these links are still lacking, a strong rationale suggests that dietary compounds and patterns may be related to pathways involved in inflammation and autoimmunity in RA [13]. Growing experimental and clinical evidence suggests that gut dysbiosis may induce a chronic inflammatory response that could be linked to RA disease development [14]. One of the proposed mechanisms whereby dietary interventions may influence RA is through the modulation of intestinal microbiota and intestinal barrier function [15]. Anti-inflammatory diets, including the Mediterranean Diet (MD), vegetarian, and vegan diets have already been shown to result in a significantly lower subjective pain rating by RA patients, when compared to ordinary diets [16].

OA is the most prevalent joint disease in the world with a tendency to increase further due to the aging population and growing rates of obesity [17]. OA is considered a complex multifactorial disease that affects the whole joint and can be classified as primary (or idiopathic) and secondary, such as in the context of trauma, surgery, and abnormal joints at birth, among others [18]. Chronic, low-grade inflammatory processes in OA contribute to disease progression [19]. In OA, biomechanical cartilage injury and joint inflammation compromise the viability of the chondrocyte, reprogramming viable chondrocytes to proinflammatory and pro-catabolic responses [19]. Secreted inflammatory molecules such as proinflammatory cytokines are important mediators of the disturbed mechanisms underlying OA pathophysiology. In particular, interleukin (IL)-1β and tumor necrosis factor (TNF) manage the degeneration of the articular cartilage matrix [20]. Gut microbiota, strongly modulated by the host’s diet, is reported to be implicated in the initiation and progression of inflammation-driven diseases; moreover, it has emerged as a risk factor for the production of proinflammatory cytokines and bacterial metabolites, which may be involved in OA onset and evolution [21,22]. The role of dietary factors and patterns in the susceptibility and progression of OA has been explored as a possible adjuvant non-pharmacological strategy to impact the natural history of the disease [23,24].

Regarding FM, a chronic condition characterized by widespread pain, along with various other factors, evidence implicating inflammation in the disease pathogenesis has been increasing [25]. IL-6 and IL-8 were shown to be elevated in FM patients, and both cytokines were correlated with clinical scores [26]. Evidence for alterations in the gut microbiome in FM patients was reported, and in fact, using machine-learning algorithms, the microbiota composition alone allowed for the classification of FM patients and controls [27]. With respect to diet, similarly to RA and OA, plant-based diets including vegetarian or vegan diets have been suggested to exert beneficial effects in FM. Nadal-Nicolás and collaborators conducted a systematic review of these dietary patterns and reported significant improvements in biochemical parameters (total cholesterol, peroxidases, and fibrinogen), quality of life, quality of sleep, pain at rest, and general health status in FM patients [28].

Finally, not only whole foods and dietary patterns have been investigated in RDs but also their isolated nutrients and/or bioactive compounds. Research on the use of food extracts has been emerging due to the growing interest in alternatives to non-steroidal anti-inflammatory agents in the management of chronic inflammation [29], including in RDs. Relevant to our discussion, regarding the MD, it has been emphasized that there are specific components of this dietary pattern, besides the whole foods that are most frequently highlighted in this diet, that may also greatly contribute to the observed health benefits, such as herbs and spices and their bioactive properties [30].

## 3. Insights into the Bioactive Properties of Herbs and Spices and Proposed Mechanisms Whereby They May Be of Interest in Rheumatology

Beyond their known culinary properties, herbs and spices have been investigated from a health perspective. Their therapeutic potential has been investigated for several diseases, including RDs [9], cancer [31], diabetes mellitus [32], cardiovascular [33], gastrointestinal [34], and neurodegenerative diseases [35]. Increasing evidence highlights their richness in bioactive molecules such as sulfur-containing compounds, tannins, alkaloids, phenolic diterpenes, and vitamins, especially flavonoids and polyphenols, together with their antioxidant, anti-inflammatory, antitumorigenic, and anticarcinogenic properties [36,37,38].

Cinnamon (*Cinnamomum zeylanicum*) belongs to the Lauraceae family and contains a variety of bioactive compounds, such as cinnamon aldehyde (CA), cinnamic alcohol, cinnamic acid, and cinnamate, that can affect health in different forms [39]. Apart from arthritis, the effects of cinnamon on various chronic diseases have been explored, including in diabetes, arteriosclerosis, and Alzheimer’s disease [40]. Regarding the mechanisms whereby cinnamon may exert its effects, Reddy and collaborators [41] reported that cinnamon had an inhibitory effect on lipopolysaccharide (LPS)-induced nuclear factor kappa-light-chain-enhancer of activated B cells (NF-kB) transcriptional activity. This is an important role, since NF-kB is a transcription factor known for regulating the expression of proinflammatory genes. Moreover, recent studies indicate that cinnamon, as well as its metabolite, sodium benzoate, can upregulate regulatory T cells (Tregs) and T helper (Th) 2 cells, suppress autoimmune Th17 and Th1, inhibit inflammatory infiltration, decrease the expression of proinflammatory molecules and, thus, might have therapeutic significance in autoimmune diseases [42]. 

Curcumin, a major curcuminoid derived from the extract of the rhizome of *Curcuma longa* (turmeric), is a polyphenol compound that was demonstrated both in vitro and in vivo to have significant anti-inflammatory effects [43]. Curcumin has a wide range of molecular targets, namely transcription factors, growth factors and their receptors, cytokines, enzymes, and genes regulating cell proliferation and apoptosis, but its poor bioavailability, attributed to limited absorption, fast metabolism, and rapid systemic elimination, is highlighted as a major concern [44]. Of interest, curcumin bioavailability was shown to be enhanced when consumed as fresh or powdered turmeric, which could be due to the existence of other turmeric compounds and/or a turmeric matrix effect [45]. Evidence indicates that curcumin exerts its effects by interfering with the arachidonic acid metabolism, namely by binding to proteins such as cyclooxygenase (COX)-2 and lipoxygenase [46,47]. The mechanism of action of curcumin was reported to be, therefore, similar to the mechanism of non-steroidal anti-inflammatory drugs (NSAIDs) [46], commonly used in RDs. A recent systematic review and meta-analysis of 31 Randomized Controlled Trials (RCTs) that evaluated the effect of curcumin and *Curcuma longa* extract in the treatment of autoimmune diseases demonstrated that curcumin decreased disease activity in RA (CRP) [48]. 

Garlic (*Allium sativum*) and its derivatives have been extensively studied in in vitro and in vivo animal models to assess its anti-inflammatory and immunomodulatory properties [49]. Mechanistically, garlic seems to enhance the functioning of the immune system by stimulating macrophages, lymphocytes, natural killer (NK) cells, dendritic cells, and eosinophils through modulation of cytokine secretion, immunoglobulin production, phagocytosis, and macrophage activation [49]. Garlic organosulfur compounds (GOSCs), including *γ*-glutamyl-S-allyl-L-cysteines (GSACs), alliin, S-allylcysteines (SACs), diallyl sulfide (DAS), diallyl disulfide (DADS), diallyl trisulfide (DATS), and allicin, are thought to be responsible for the biological activities of garlic [50]. Among the GOSCs, DATS stands out as a potential anti-inflammatory agent in both cell and animal models, by downregulating the NF-kB pathway, which leads to the inhibition of the transcription of several cytokine genes that take part in proinflammatory responses [50,51]. DAS was also highlighted [52] as a potential phytochemical to inhibit TNF-α and histamine-induced inflammation by reducing the levels of reactive oxygen species (ROS) formation. Regarding its effects on humans, a systematic review and meta-analysis of RCTs on the effects of garlic on inflammatory biomarkers, which included a total of sixteen studies, concluded that garlic supplementation (doses ranged from 12 to 3600 mg/day, and intervention duration ranged from 2 to 52 weeks) significantly decreased serum concentrations of C-reactive protein (CRP), IL-6, and TNF-α [53].

*Zingiber officinale* Roscoe, from the Zingiberaceae family, is the fresh root of ginger and has a complex chemical composition, including more than 300 types of species [54]. The consumption of ginger appears to be safe and potentially beneficial for human health and well-being, with the greatest confidence in its antiemetic effects, analgesic effects in OA, and glycemic control [55]. Of interest to RDs, ginger extract was found to suppress inflammation by decreasing prostaglandin E2 and nitric oxide production in a rat adjuvant arthritis model [56]. Additionally, the phenolic substance 6-gingerol, a major principle of ginger, was suggested to act as an anti-inflammatory agent by blocking NF-kB and protein kinase C (PKC) signaling, two major proinflammatory signaling cascades [57]. Interestingly, in human intestinal epithelial cells, 6-shogaol (during the drying process, gingerols dehydrate to shogaols) was shown to inhibit the TNF-α-induced barrier disturbance, which may suggest a protective role of ginger against barrier dysfunction in intestinal inflammation [58].

Saffron, derived from the *Crocus sativus* L. flower, and its constituents (safranal, crocin, and crocetin) could represent an adjuvant therapy for various conditions and are especially promising in patients with RDs, as they may modulate inflammation, antioxidant status, depression, anxiety, and pain [59]. In vitro and in vivo findings state that saffron and its main constituents show anti-inflammatory and immunomodulatory properties through both modulation of innate immunity (neutrophils, macrophage, and natural killer cells) and modulation of acquired immunity (B cells, Th1/Th2 balance, and inflammatory and anti-inflammatory cytokines) [60]. A recent systematic review and meta-analysis [61] reported that saffron supplementation did not have a significant effect on serum levels of CRP, TNF-α, and IL-6, which are inflammatory biomarkers. Nevertheless, in a subgroup analysis, saffron was reported to significantly decrease CRP and TNF-α serum concentrations in some subpopulations [61]. Similarly to the other herbs and spices previously mentioned, there is currently not enough evidence to make recommendations, and more research is necessary to define the proper dose and conclude with certainty the efficacy of saffron in RD-related outcomes [59].

Lastly, on this subject, it is crucial to consider that the bioactive properties mentioned above may be affected by storage or cooking methods. Chohan et al. [62] investigated the impact of cooking and storage on the antioxidant capacity of common culinary herbs and spices, including cinnamon and ginger, among other herbs and spices. They found that their antioxidant capacity significantly increased with simmering, soup making, and stewing, whilst grilling and stir frying reduced it [62]. Moreover, freezing herbs (at −20 °C) and cold maceration had preservative effects on antioxidant capacity [62]. Hence, the promotion of their consumption, if not through a dietary supplement, must consider these factors and empower patients to be able to use and accommodate herbs and spices appropriately in order to maintain their bioactive properties as much as possible.

Although different herbs and spices contain distinct bioactive compounds, with distinct chemical arrangements, they appear to exhibit similar effects on the human body, leaving room for discussion on possible common signaling/structure between them. To the best of our knowledge, these dietary compounds do not have single targets or receptors in the human body, but they seem to share similarities that may explain why different herbs/spices may act similarly. As previously mentioned, ginger, garlic, and cinnamon have all been found to inhibit the activation of NF-kB, thus attenuating inflammatory responses. In addition, these bioactive compounds seem to be able to regulate the production of cytokines involved in inflammation, such as TNF-a and IL-6.

Furthermore, phenolic compounds are a common feature of all the herbs and spices mentioned above. Plant phenolics include compounds such as simple phenols, phenolic acids (the principal polyphenols produced by plants), and flavonoids [63]. Among others, cinnamon has been shown to contain the phenolic compounds epicatechin and catechins [64], turmeric contains the polyphenol curcumin [65], garlic contains polyphenolic compounds such as hydroxybenzoic acids and flavanols [66], ginger contains the phenolic compound gingerol [67], and lastly, phenolic compounds including catechol, vanillin, and cinnamic acid were found in saffron [68]. Phenolic compounds are characterized by the presence of one or more benzene rings and hydroxyl groups and are known for their antioxidant properties [69]. Several mechanisms have been proposed for the antioxidant capacity of phenolic compounds, but radical scavenging via hydrogen atom donation is believed to be the main mechanism [70]. When looking at the molecular structure of these herbs/spices, it is worth noting that the common features between these compounds lie in the presence of benzene rings and the presence of conjugated systems of double bonds. Benzene rings are chemical compounds made up of six carbon atoms and three double bonds connected in a hexagonal ring [71]. A conjugated system is a system of connected p-orbitals with delocalized electrons in compounds with alternating single and double bonds, which can lower the overall energy of the molecule and increase its stability [72]. The presence of these features allows these herbs/spices to scavenge free radicals and neutralize ROS, which contributes to their anti-inflammatory and antioxidant properties [73]. Free radicals are highly reactive molecules that can cause damage to cells and tissues by inducing oxidative stress and promoting inflammation [74]. Scavenging free radicals aids in preventing ROS formation and balances oxidative stress, thereby lowering the inflammatory cascade [74]. This may be a common mechanism contributing to the potential health benefits of herbs and spices mentioned throughout this review.

Of interest, polysaccharides are another common feature among some herbs and spices and were described to be present in cinnamon [75], turmeric [76], garlic [77], ginger [67], and saffron [78]. Interestingly, the human body is not able to properly assimilate polysaccharides, leaving most to be fermented by gut microbiota [79]. Gut microbiota modulation by herbs and spices will be further discussed in the next section of this manuscript.

### Gut Microbiota Modulation by Herbs and Spices

Relevant to our discussion, one of the proposed mechanisms for the potential effects of herbs and spices on human health is through gut microbiota modulation. Evidence suggests that substrates present in herbs and spices can drive favorable changes in gut communities and contribute to their proposed health-related effects [80]. Polyphenols, one of the bioactive molecules of herbs and spices mentioned previously, are proposed to confer at least part of their health benefits via modulation of gut microbiota composition and function [81].

Interestingly, all the spices mentioned above have been investigated for their effects on gut microbiota. For instance, both cinnamon and ginger were shown to be able to enhance intestinal barrier function and modulate gut microbiota in animal models [82,83]. Studies have also highlighted a bidirectional interaction between curcumin and its metabolites and microbiota, that is, the regulation of gut microbiota by curcumin and the biotransformation of curcumin by the gut microbiota [84]. Garlic contains prebiotic components such as fructans, antibacterial compounds, and organosulfur compounds and was shown in a mouse model to ameliorate the induced dyslipidemia and disturbance of gut microbiota of a high-fat diet [85]. Banskota et al. [86] suggested that saffron exerts its effects by inhibiting proinflammatory cytokine secretion and preserving gut microbiota diversity in a mice model of inflammatory bowel disease. Moreover, it was found that saffron-treated mice showed an increase in short-chain fatty acids (SCFAs), such as isobutyric acid, acetic acid, and propionic acid, vital for the regulation of intestinal epithelial cells [86].

In humans, Lu et al. [87] investigated the effects of 5 g capsules containing mixed spices at culinary doses (1 g (20%) cinnamon, 1.5 g (30%) oregano, 1.5 g (30%) ginger, 0.85 g (17%) black pepper, and 0.15 g (3%) cayenne pepper) on gut microbiota and SCFA production over 2 weeks in healthy adults [87]. Compared to the control group, spice consumption resulted in a significant reduction in Firmicutes phylum abundance [87]. The production of individual fecal SCFAs was not significantly changed by spice consumption in this study. Nevertheless, mixed spice consumption significantly modified gut microbiota, further suggesting a prebiotic effect of spice consumption [87]. Khine also investigated the effects of mixed spices (Indian curry) consumption on the gut microbiome and found that a single serving of mixed spices could significantly modify/restore gut microbiota, as compared with a control diet which was low in polyphenols [88].

**Figure 1:**
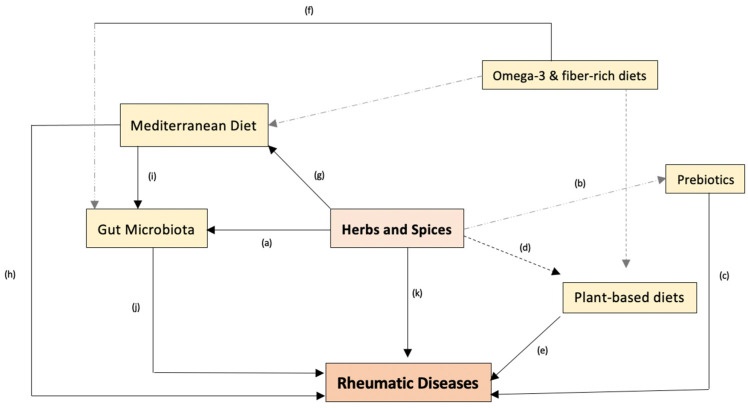
The proposed interplay between dietary interventions and RDs. (a) Culinary herbs and spices have been recognized for their potential to positively promote microbial modulation by stimulating the production of SCFAs during fermentation, (b) thereby exhibiting a prebiotic effect [90]; (c) studies have shown probiotic supplementation to improve disease activity and the inflammatory status of RA patients when used in combination with the patient’s pharmacological therapy [91]. (d) Herbs and spices are frequently used in healthy plant-based (including the MD) or vegan cooking to enhance food flavor; (e) studies have shown improvements in RA [92], FM [93], and OA [94] symptoms and inflammatory biomarkers with plant-based diets. (f) Dietary fiber found in these plant-based diets can improve gut bacteria composition and increase bacterial diversity in RA patients, thus reducing their inflammation and joint pain [92]. Accumulating evidence has shown omega-3 polyunsaturated fatty acids (PUFAs) to exert profound effects on the intestinal microbiota; in particular, eicosapentaenoic acid (EPA) and docosahexaenoic acid (DHA) have been shown to suppress inflammation and exert a beneficial effect on a variety of inflammatory-related diseases, such as RA and potentially other RDs [95]. (g) As mentioned previously, spices and herbs are an important component of the MD. This dietary pattern has been extensively studied in many inflammatory diseases, including in RDs. (h) Adherence to the MD has shown improvements in disease activity, quality of life, pain, and stiffness in RA patients [96]. (i) Evidence suggests that the gut microbiota of individuals who follow a MD-style pattern significantly differs from that of subjects who follow a western food pattern. The MD can modulate the gut microbiota by increasing its diversity and changing the proportion of specific bacteria, leading to a higher production of SCFAs, thus contributing to the reduction in the inflammatory status of subjects [97]. On the other hand, microbial dysbiosis (j) has been strongly linked as a potential causative factor of systemic autoimmune diseases, including AR, OA, and FM [98,99,100]. This is because gut microbes directly interact with the immune system by a variety of mechanisms, being that these interactions modulate crucial immune functions that can be pivotal in the pathogenesis of RDs [101]. (k) As shown throughout this review, the effect of spices and herbs on RDs can be promising through various proposed mechanisms. Despite the limitations of the available RCTs, the whole interplay represented above explores this promising effect.

Overall, these findings highlight the importance of exploring the role of gut microbiota as a mechanism whereby dietary interventions may be relevant in RDs. As stated above, gut microbiota, among other factors, may play a role in the initiation and progression of inflammation-driven diseases. The interplay between gut microbiota and the host immune system is reported to be relevant for balancing and resolving inflammation and may represent a potential therapeutic strategy [89]. Figure 1 illustrates the rationale behind the interest in herbs and spices in relation to RDs through various dietary interventions and through gut microbiota modulation, while also highlighting other diet-related components that have been investigated as potentially beneficial for RDs.

## 4. Experimental Evidence from Human Studies on Herbs and Spices in RDs

### 4.1. Rheumatoid Arthritis

With growing experimental evidence on the potential of herbs and spices in inflammation, a variety of human trials have been conducted in the last decades. To the best of our knowledge, their effects on RA patients were investigated in a total of 10 clinical trials, with the following herbs and spices: cinnamon [102], curcumin [103,104,105,106,107], garlic [108,109], ginger [110,111], and saffron [112,113]. Table 1 shows the characteristics and main findings of these trials.

Shishehebor et al. [102] conducted an RCT on the effect of *Cinnamomum burmannii* supplementation (four daily capsules, 500 mg) for 8 weeks in women with established RA that were under treatment with DMARDs but not receiving NSAIDs or cytokine inhibitors [102]. Compared to the control group, the authors found that cinnamon intake significantly reduced the disease activity score-28 (DAS28) (6.04 ± 0.52 at baseline to 3.92 ± 0.52 at the end of the study; *p* < 0.001). Moreover, the intervention resulted in a significant reduction in the Visual Analogue Scale (VAS) for pain, tender joint count (TJC), and swollen joint count (SJC), as well as a significant decrease in serum CRP and TNF-a levels. This intervention proved to be safe and potentially beneficial to improve disease activity, inflammatory markers, and clinical symptoms in patients with RA [102].

Regarding the effects of curcumin supplementation in RA patients with high disease activity (DAS28 > 5.1), Chandran et al. carried out a three-arm study, with patients receiving curcumin (500 mg/day) and diclofenac sodium (50 mg/day) alone or their combination twice daily for 8 weeks [103]. Patients taking DMARDs or other antiarthritic therapy were excluded. Significant improvements in DAS28 were reported in all the arms. Of interest, the curcumin group showed the highest percentage of improvement of DAS28. Patients who received curcumin achieved higher American College of Rheumatology (ACR) response rates than the other two groups. The components of ACR response are SJC, TJC, patient’s global assessment (GA), physician’s GA, disability index, and health assessment questionnaire (HAQ). Differences between groups were found for CRP, which showed significant improvement in the curcumin group only. Although the results are promising, the fact that this trial was an open-label study represents a major limitation [103]. 

A novel hydrogenated curcuminoid formulation was also investigated in a three-arm RCT in which RA patients were randomized to receive a 250 mg or 500 mg curcumin capsule or placebo daily over a period of three months [106]. Compared to the placebo, after intervention, improvements were observed in all assessed efficacy measures including DAS28 (4.39 ± 0.16 at baseline to 2.16 ± 0.62 at the end of the study in the lower dose group; 4.42 ± 0.61 at baseline to 1.59 ± 0.37 at the end of the study in the higher dose group), VAS scores for pain, ESR, CRP, and RF [106]. The comparative efficacy of two different doses of curcumin (low dose of 250 mg or high dose of 500 mg of curcumin twice daily) with a placebo, for 90 days, in active RA patients was also investigated by Amalraj et al. [104]. In this trial, capsules were completely constituted of a natural turmeric matrix with no excipients/additives. Both intervention groups showed a significant improvement in DAS28 (4.51 ± 0.64 at baseline to 2.14 ± 0.16 at the end of the study in the low-dose group; 5.29 ± 0.54 at baseline to 1.80 ± 0.36 at the end of the study in the high-dose group), while there were no significant changes in the placebo group. Significant improvements were also reported for VAS scores for pain, ESR, CPR, and RF values in patients in the intervention group [104].

Contradicting the previous results, in the RCT conducted by Javadi et al. [105], no significant changes were observed for DAS-28, TJC, SJC, and ESR between the intervention and the control groups. In this trial, the intervention consisted of curcumin nanomicelle capsules (40 mg) three times a day for 12 weeks, in addition to the patients’ current medications [105]. Finally, Pourhabibi-Zarandi et al. [107] evaluated the effects of curcumin supplementation (500 mg once a day) or placebo for 8 weeks on metabolic, inflammatory, and anthropometric factors in women with moderately active disease (DAS28 score between 3.2 and 5.1). At the end of the study, the intervention significantly decreased ESR, high sensitivity CRP (hs-CRP), homeostatic model assessment for insulin resistance (HOMA-IR), body weight, body mass index, and waist circumference of patients compared with the placebo. Although this RCT did not address relevant clinical parameters such as disease activity, it suggests curcumin efficiency, as a part of an integrated approach, to modulate metabolic factors, inflammation, and adiposity [107].

As for the effects of garlic supplementation (1000 mg powder capsules, equivalent to 2.5 g of fresh garlic), Moosavian et al. [108,109] conducted an RCT with women with moderate-to-severe RA (DAS28 > 3.2). In this trial, DAS28, pain VAS, TJC, SJC, fatigue, and serum levels of CRP and TNF-a decreased significantly in the garlic group compared with the placebo group. Furthermore, at the end of the study, there was a significant increase in serum levels of total antioxidant capacity (TAC) and a significant decrease in malondialdehyde (MDA) in the garlic group, when compared with the placebo group. Pain VAS and HAQ scores also decreased in the garlic group compared with the placebo. Unlike some of the results with curcumin supplementation, the changes in ESR values were not statistically significant [108,109].

The effects of supplementation with 1500 mg/day of ginger powder in immunological and inflammatory markers and in some immunity and inflammation intermediate gene expressions was investigated by Aryaeian et al. [110,111] in patients with active RA. Ginger supplementation significantly decreased DAS28, hs-CRP, and IL-1β. Although TNF-α levels were reduced in the intervention group, no significant differences were found between the two groups. As for gene expression, a statistically significant decrease in retineic-acid-receptor-related orphan nuclear receptor gamma (RORγt) and T-bet gene expression and a significant increase in forkhead box P3 (FoxP3) gene expression were observed. This study sheds light on the possible mechanisms by which ginger may affect RA disease activity, namely through the improvement in immune system function by decreasing RORγt and T-bet gene expression as factors involved in inflammation and autoimmunity and by increasing FoxP3, a factor involved in tolerance [110,111].

Lastly, saffron supplementation was studied by Hamidi et al. [112] and Sahebari et al. [113]. Hamidi et al. [112] evaluated the effects of 100 mg daily of a saffron supplement for 12 weeks on clinical outcomes and inflammatory and oxidative markers in female patients with active RA. In this trial, saffron supplementation significantly decreased DAS28 (5.09 ± 1.10 at baseline to 4.33 ± 0.94 at the end of the study), TJC, SJC, and pain VAS at the end of intervention between the two groups and in the saffron group compared with baseline values. Physician GA and ESR were significantly decreased after intervention. Regarding inflammatory and oxidative markers after the intervention, TNF-α, interferon gamma (IFN-γ), hs-CRP, and MDA levels decreased and TAC increased, but differences were not significant between the two groups [112]. Sahebari et al. evaluated the same dose of saffron over the same timeframe (100 mg/day for 12 weeks) but on newly diagnosed RA patients without previous treatment [113]. Standard therapy, including prednisolone, oral methotrexate, folic acid, vitamin D, calcium, and alendronate, was administered similarly in both groups, but the intervention group received the saffron pill in addition to standard therapy. At the end of the study, differences were not seen for DAS28 and HAQ scores between the intervention and control group, meaning that saffron supplementation in addition to standard therapy did not significantly improve disease activity and functional status compared to patients treated using standard pharmacological therapy [113]. 

Altogether, there is some evidence from human trials that herbs and spices may play a role in improving disease activity and the immunological and inflammatory status of RA patients. Nevertheless, the studies have conflicting results. Although it is not possible to draw definite conclusions, as of now, curcumin is the most widely studied spice in RA with promising results, with doses ranging from 250 to 500 mg per day in most studies, and should be further investigated in well-conducted and reproducible intervention trials.

**Table 1:** Effects of herbs and spices on Rheumatoid Arthritis in clinical trials.

Herb/Spice	Author, Year, and Country	Study Design	Population, Sample Size (Intervention, Control)	Preparation/Dose (Intervention vs. Control) and Duration	Evaluated Parameters	Main Findings	Reported Adverse Effects (AEs)
Cinnamon	Shishehbor et al. [102], 2018, Iran	Double-Blind RCT	36 women with active RA (18 patients/arm)	4 capsules of either 500 mg cinnamon powder or placebo daily for 8 weeks	DAS-28, pain VAS, SJC, TJC, ESR, CRP, TNF-a	↓DAS-28, ↓pain VAS, ↓SJC, ↓TJC, ↓CRP, ↓TNF-a	Intervention group: 1 patient reported mild gastric discomfort
Curcumin	Chandran et al. [103], 2012, India	Randomized single-blind pilot study	45 RA patients (15 patients/arm)	500 mg curcumin or 500 mg curcumin + 50 mg diclofenac sodium vs. 50 mg diclofenac sodium twice daily for 8 weeks	DAS-28, disease activity VAS, pain VAS, SJC, TJC, ESR, HAQ, CRP	↓DAS28, ↓SJC, ↓TJC, ↓disease activity VAS, ↓pain VAS, ↓HAQ, ↓ESR	Diclofenac sodium: 3 adverse events. Curcumin + diclofenac sodium: 1 adverse event; curcumin: 2 adverse events
	Amalraj et al. [104], 2017, India	Double-Blind RCT	36 patients with active RA (12 patients/arm)	250 mg or 500 mg curcumin vs. 500 mg of food-grade starch twice/day for 12 weeks	DAS28, pain VAS, TJC, SJC, ESR, CRP, RF	↓DAS28, ↓SJC, ↓TJC, ↓pain VAS, ↓ESR, ↓CRP, ↓RF	None
	Javadi et al. [105], 2019, Iran	Double-blind RCT	65 RA patients (*n* = 30, *n* = 35)	40 mg curcumin nanomicelle vs. 500 mg wheat flour 3×/day for 12 weeks	DAS-28, TJC SJC, ESR	No significant changes were observed between groups	None
	Jacob et al. [106], 2019, India	Double-blind RCT	24 patients with active RA (8 patients/arm)	250 mg/day or 500 mg/day curcumin vs. placebo (non-described) for 12 weeks	DAS28, pain VAS, ESR, CRP, RF	↓DAS28, ↓pain VAS, ↓ESR, ↓CRP, ↓RF	None
	Pourhabibi-Zarandi et al. [107], 2022, Iran	Double-blind RCT	48 women with moderately active RA (24 patients/arm)	500 mg/day curcumin vs. 1 capsule/day of starch flour for 8 weeks	ESR, hs-CRP, lipid profile, glycemic and anthropometric indices	↓ESR, ↓hs-CRP	None
Garlic	Moosavian et al. [108,109], 2020, Iran	Double-blind RCT	70 women with moderate-to-severe RA (35 patients/arm)	1000 mg/day garlic powder vs. 500 mg starch for 8 weeks	DAS28, pain VAS, SJC, TJC, HAQ, ESR, CRP, TNF-a, TAC, MDA	↓DAS28, ↓pain VAS, ↓SJC, ↓TJC, ↓HAQ, ↓CRP, ↓TNF-a, ↑TAC, ↓MDA	Intervention: 1 patient with stomach pain
Ginger	Aryaeian et al. [110,111], 2019, Iran	Double-blind RCT	66 patients with active RA (*n* = 33, *n* = 30)	1500 mg/day ginger vs. roasted wheat flour for 12 weeks	DAS-28, hs-CRP, IL-1β, IL-2 and TNF-α, gene expression of NF-κB, PPAR-γ, FoxP3, T-bet, GATA-3, and RORγt	↓DAS28, ↓hs-CRP, ↓IL-1β, ↑ FoxP3, ↓T-bet, ↓RORγt,	NA
Saffron	Hamidi et al. [112], 2019, Iran	Double-blind RCT	66 women with active RA (33 patients/arm)	100 mg/day saffron vs. 100 mg/day hydroxy propylmethyl cellulose for 12 weeks	DAS28, pain VAS, morning stiffness, SJC, TJC, PGA, TNF-α, IFN-γ, hs-CRP, ESR, MDA, TAC	↓DAS28, ↓SJC, ↓TJC, ↓pain VAS	Intervention: stomach pain (*n* = 1) Control: stomach pain (*n* = 1)
	Sahebari et al. [113], 2020, Iran	Double-blind RCT	55 newly diagnosed RA patients (*n* = 28, *n* = 27)	100 mg saffron (with additives: starch, lactose monohydrate, starch sodium glycolate, PVP K30) vs. pills with the mentioned additives for 12 weeks	DAS28, VAS, pain score, TJC, SJC, HAQ	No changes were observed between groups	None

Symbols: ↑, increased; ↓, decreased. Abbreviations: disease activity score-28 (DAS28), erythrocyte sedimentation rate (ESR), forkhead box P3 (FoxP3), GATA-binding protein 3 (GATA-3), health assessment questionnaire (HAQ), high-sensitivity C-reactive protein (hs-CRP), interferon gamma (IFN-γ), interleukin (IL), malondialdehyde (MDA), nuclear factor kappa-light-chain-enhancer of activated B cells (NF-kB), visual analog scale (VAS), tender joint count (TJC), total antioxidant capacity (TAC), tumor necrosis factor alpha (TNF-α), peroxisome proliferator-activated receptor-gamma (PPAR-γ), retineic-acid-receptor-related orphan nuclear receptor gamma (RORγt), rheumatoid factor (RF), swollen joint count (SJC), physician global assessment (PGA).

### 4.2. Osteoarthritis

Current therapy for OA mainly consists of analgesics, NSAIDs, and cortisone, which are able to control pain and inflammation, but are associated with a broad spectrum of adverse effects, drug interactions, and contraindications [114]. Spices have been increasingly studied in human trials as a natural treatment option for inflammatory and pain-related symptoms in patients suffering from OA. Table 2 details the characteristics and main findings of human trials that investigated the effects of herbs and spices in OA.

Curcuminoids are natural polyphenols with strong antioxidant capacity and, thus, have been increasingly studied in the management of OA, as oxidative stress is implicated in the pathogenesis of the disease. The effect of curcumin (*Curcuma domestica* extract) supplementation (1500 mg/day) was compared with ibuprofen (1200 mg/day) in patients with knee OA for 4 weeks in the RCT conducted by Kuptniratsaikul et al. [115]. Compared to baseline, decreased scores were observed for all assessed outcome measures, including WOMAC total and its subscales, pain, stiffness, and physical function limitations, in both groups. Although no between-group difference was seen, this intervention suggests that *Curcuma domestica* extracts were as efficacious as ibuprofen in reducing pain and functional improvement [115]. In addition, the rate of GI symptoms was significantly lower in the curcumin group than in the ibuprofen group (10.8% vs. 18.1%). 

Supplementation with *Curcuma longa* (500 mg 2×/day) for 4 months was also studied by Srivastava et al. [116] to evaluate its effects on inflammatory and oxidative stress in patients with OA. Compared to the placebo, knee pain by VAS and WOMAC scores for pain, stiffness, and physical function limitations were significantly reduced in the curcumin group when compared with baseline values. Moreover, levels of inflammatory biomarkers, including IL-1β, ROS, and MDA, were significantly reduced in the curcumin group at the end of the study (65.61 ± 21.59 to 21.11 ± 1.176, 2553 ± 775.67 to 1200 ± 864.08, and 3.85 ± 0.12 and 3.69 ± 0.12, respectively) [116]. The clinical improvement in WOMAC score and VAS may be subjective, but the levels of biomarkers are objective parameters to evaluate the inflammatory status and oxidative stress. It is widely established that there is an increase in these markers in many inflammatory disorders, and the lowering of these strongly correlates with disease activity [117]. Similarly, a study evaluating the efficacy of curcumin supplementation combined with piperine in reducing oxidative stress found consistent results. Forty patients were randomized into a curcuminoid group (500 mg 3×/day) or a matched placebo capsule group for a period of 6 weeks. At the end of the trial, there was a significant elevation in serum activities of superoxide dismutase (SOD), an improvement in serum glutathione (GSH), and a reduction in MDA concentration in the curcuminoid group. Between-group comparison showed greater efficacy of curcuminoids compared with the placebo in improving oxidative stress biomarkers [118].

Curcumin supplementation for 12 weeks was studied in an RCT comparing the efficacy of extracts containing the combination of boswellic acid and curcumin (Curamin, 500 mg/day), curcumin alone (500 mg/day), or placebo in the treatment of OA symptoms [114]. Patients did not receive NSAIDs, analgesics, glucocorticoids, nor any other medication for joints or cartilage. Compared to the placebo, significant improvements were observed in both curcumin groups in all the WOMAC assessed parameters, as well as in all the physical performance measures (PPMs) including pain on standing from a chair (via 30 s chair stand repetitions), pain on climbing stairs (via the stair climb test), 40 m walking speed, and functional mobility (via the TUG test). Serum ESR and CRP levels did not change significantly, but a significant between-group difference was seen when comparing the Curamin and placebo groups after only 12 weeks of treatment. The effect size of these results compared to placebo was comparable for both treatment groups but was bigger in the *Curcuma longa* and *Boswellia serrata* extracts in combination, presumably due to synergistic effects [114]. As far as we know, the most recent study of spice supplementation in OA focuses on the effect of a curcumin extract (1000 mg/day) on knee pain [119]. Over 8 weeks, the Knee Injury Osteoarthritis Outcome Score (KOOS) pain subscale, knee pain ratings, and the total Japanese Orthopedic Association Score for Osteoarthritic Knees (JOA) improved more in the curcumin group compared to the placebo group. In the curcumin group, there were significant improvements in the 30 s chair test, TUG test, and 6 min walk test but not the 40 m FPWT. The Patient-Reported Outcomes Measurement Information System-29 (PROMIS-29) was also assessed, but no significant differences were found. A significant difference was observed in medication intake, since pain-relieving medication use decreased in 37% and 13% of participants in the curcumin and placebo groups, respectively. The KOOS is an extension of the WOMAC OA index designed to assess patient-relevant outcomes associated with knee injury and pain [120]. The JOA is a validated subjective knee scoring tool developed for the assessment of functional status in patients with knee OA [121]. Lastly, the PROMIS-29 is a validated generic health-related quality of life self-report survey [122].

Saffron has also been widely studied for its anti-inflammatory effects in OA. The immunoregulatory effects of Krocina, a water-soluble carotenoid made from saffron extract, were studied in an RCT in OA patients for 4 months. This trial demonstrated that Krocina at a dose of 15 mg/daily can meaningfully reduce CRP levels (2.92 ± 0.55 mg/L vs. 1.77 ± 0.26 mg/L), percentage of Th17 cells (6.93 ± 0.6 vs. 5.09 ± 0.5), and geometric mean fluorescence intensity (GMFI)-IL-17 (5.73 ± 0.48 vs. 4.29 ± 0.36); the same was not seen in the placebo group. At the end of trial, the percentage of Treg cells and Treg/Th17 cell ratio showed a meaningful increment in the consumption of Krocina group but not in the placebo group. Additionally, the numbers of GMFI-FOXP3, CD4+ Th cells, and CD8+ T cells were not significantly changed in both groups [123]. Taken together, this trial concludes that Krocina has potential anti-inflammatory and immunoregulatory effects on patients with OA; however, further research is needed with a control group not receiving NSAIDs to draw a better conclusion regarding the above effects.

Garlic has been studied in many autoimmune and inflammatory diseases, such as OA, for its potential health benefits. Apart from NSAIDs, the management of knee OA includes an interconnection between patient education, physiotherapy, quadricep strength training, and weight reduction [124]. An RCT conducted by Hussein et al. compared the effect of a comprehensive rehabilitation program versus a combined garlic therapy (900 mg/daily) in managing the clinical manifestations and quality of life in patients with knee OA [124]. After 8 weeks of intervention, there was a significant decline in synovial cytokines, including IL-1β, IL-6, and TNFα, only in the garlic group. This was accompanied by better control of knee pain and effusion, as well as better muscular strength and quality of life. Significant differences were seen, highlighting the beneficial effect of garlic regarding the control of inflammation in knee OA. The effect of garlic supplementation (1000 mg/day) on symptom relief was studied in overweight or obese women with knee OA. Following the 12-week supplementation period, WOMAC total score and its subscales were significantly improved only in the garlic group, except for the pain subscale, which was also decreased in the placebo group. However, changes in WOMAC parameters showed no statistically significant differences between the two groups [125]. The same authors published an additional article with the same study design and sample but evaluating the effects of garlic supplementation on the proinflammatory adipocytokines, resistin, TNF-α, and pain severity. Post-intervention, serum concentrations of resistin were significantly decreased in the garlic group, no significant changes in TNF-α were observed in either of the groups, and lastly, pain scores were significantly decreased in the garlic group but not in the placebo group [126]. A few years later, the same authors studied the efficacy of garlic supplementation on knee OA symptoms. At the end of the trial, all clinical outcomes, including WOMAC total score and its subscales as well as VAS joint pain, were remarkably improved in the garlic group versus the placebo. However, no statistically significant differences were observed between groups [127]. 

A herbal formulation containing turmeric extract (300 mg of curcumin), black pepper (3.75 g of piperine), and ginger (7.5 g of gingerols) was investigated versus naproxen (250 mg), an NSAID, in an RCT [128] in which patients were randomized to receive herbal capsules (2×/day after meals) or naproxen capsules (2× in the morning and night) for 4 weeks. The plasma levels of prostaglandin E2 decreased significantly in both groups (110.53 ± 32.32 vs. 82.90 ± 22.82 for the herbal group and 102.50 ± 35.5 vs. 72.77 ± 25.66 for the naproxen group), but there were no significant differences between groups. Still, this study revealed that herbal formulations can have important effects on inflammation suppression and, hence, reinforces the potential anti-inflammatory properties of certain spices [128]. 

There are limited clinical trials evaluating the effect of ginger supplementation in patients with OA. An RCT conducted by Altman et al. [129] evaluated the efficacy and safety of a ginger extract (255 mg 2×/day) in patients with knee OA. After the 6-week treatment, pain standing was improved in both treatment groups, but the ginger group improved more than the placebo group. Pain after walking also significantly improved in the ginger group compared to the placebo group; likewise, the change in WOMAC total score was superior in the ginger group versus the placebo group, with the greatest improvement seen in WOMAC stiffness [129]. These results are in line with the results described in the few existing human trials on the use of ginger in OA [130]. A more recent pilot study evaluating the effects of ginger (25 mg) and Echinacea (5 mg) extract supplementation on inflammation and chronic pain in knee OA provides some indication of its potential benefit in inflammation; nonetheless, there are many limitations associated with this study [131]. Future clinical trials with larger sample sizes and longer follow-up periods are needed to support the observed improvements in symptoms and function. Interestingly, studies have also evaluated ginger extract in nanostructured lipid carriers as a gel/cream in OA patients, and these results have shown improvements in joint pain, physical function, and quality of life [132]. 

To our knowledge, there are no human trials investigating the effects of cinnamon on patients with OA; however, a recent study exploring the mechanism underlying the effect of CA (cinnamon) on synovial inflammation in OA showed that CA exhibited strong anti-inflammatory effects in OA fibroblasts through inhibiting the activation of the Toll-like receptor 4 (TLR4)/myeloid differentiation primary response 88 (MyD88) signaling pathway, suggesting its potential as a novel agent for the management of OA [133]. All things considered, there is some evidence from human trials that herbs and spices have a potentially beneficial role in managing OA symptoms and in the immunological and inflammatory status of OA patients. Additionally, supplementation with curcumin, saffron, ginger, and garlic seems to be well tolerated, with no serious adverse events reported. Even so, the mentioned studies have several limitations and, hence, require further confirmation in longer trials with larger sample sizes and with more stringent monitoring of medication use.

**Table 2:** Effects of herbs and spices on Osteoarthritis in clinical trials.

Herb/ Spice	Author, Year, and Country	Study Design	Population, Sample Size (Intervention, Control)	Preparation/Dose (Intervention vs. Control) and Duration	Evaluated Parameters	Main Findings	Reported Adverse Effects (AEs)
Curcumin	Kuptniratsaikul et al. [115], 2014, Thailand	Double-blind RCT	331 patients with primary knee OA (*n* = 171, *n* = 160)	1500 mg/day of *Curcuma Domestica* or 1200 mg/day of ibuprofen for 4 weeks	WOMAC *: total, pain, stiffness, and function, 6 min walk test	↓WOMAC * (total, pain, stiffness, and physical function) in both groups	Ibuprofen group: 35.7%, C. Domestica: 29.7%
	Srivastava et al. [116], 2016, India	Double-blind RCT	160 with primary knee OA (*n* = 78, *n* = 82)	500 mg *Curcuma Longa* or placebo 2×/day for 16 weeks, along with Diclofenac 50 mg/day (standard treatment as needed)	VAS, WOMAC * (pain, stiffness, and physical function), IL-Iβ, ROS, MDA	↓VAS, ↓WOMAC *, ↓IL-Iβ, ↓ROS, ↓MDA	CL group: 2, placebo group: 4
	Panahi et al. [118], 2016, Iran	Double-Blind RCT	53 patients with mild–moderate knee OA (*n* = 27, *n* = 26)	1500 mg/day of curcuminoids (combined with piperine) or placebo for 6 weeks	SOD, GSH, MDA	↑SOD activities, ↑GSH conc., ↓MDA	NA
	Haroyan et al. [114], 2018, Armenia	Double-blind RCT	201 patients with OA (*n* = 67, *n* = 66, *n* = 68)	500 mg Curamin or 500 mg CuraMed or 500 mg placebo 3×/day for 12 weeks	PPMs (30 s-CST, 40 m FPWT, TUG, SCT), WOMAC (total, pain, stiffness, physical function limitations), ESR, CRP	↓all WOMAC *, ↑30 s CST repetitions, ↓TUG time, ↓SCT, ↑40 m FPWT, ⇢⇠ESR, ⇢⇠CRP	2 in Curamin, 7 in CuraMed, 4 in placebo
	Lopresti et al. [119], 2022, Australia	Double-blind RCT	101 patients with active knee OA (*n* = 51, *n* = 50)	500 mg Curcugen or placebo 2×/day for 8 weeks	KOOS, JOA, PROMIS-29, pain, PPMs (30 s-CST, 40 m FPWT, TUG, 6-min walk test), analgesic use, AE	↓KOOS-pain, ↓pain, ↓JOA, ↑30 s CST repetitions, ↓TUG, ↓6 min walk test (m), ⇢⇠40 m FPWT, ↓pain medication use	5 in curcumin group and 10 in placebo group
Garlic	Hussein et al. [124], 2015, Egypt	Single-blind RCT	43 patients with knee OA (*n* = 15, *n* = 28)	Group 1: comprehensive rehabilitation; Group 2: garlic therapy (900 mg/day) + comprehensive rehabilitation for 8 weeks	Pain VAS, HAQ, 1-RM, BMI, IL-Iβ, IL6, TNF-α, selenium	↓Pain, ↓IL-Iβ, ↓IL-6, ↓TNF-α, ↑selenium	NA
	Salimzadeh et al. [125], 2018, Australia	Double-blind RCT	76 post-menopausal overweight or obese women with mild–moderate knee OA (*n* = 39, *n* = 37)	500 mg garlic tablet or placebo 2×/day for 12 weeks	WOMAC * (pain, stiffness, physical function limitation)	↓WOMAC * (total, pain, stiffness, physical limitation, ↓pain in placebo	NA
	Dehghani et al. [126], 2018, Iran	Double-blind RCT	76 post-menopausal overweight or obese women with mild–moderate knee OA (*n* = 39, *n* = 37)	500 mg garlic tablet or placebo 2×/day for 12 weeks	Pain VAS, resistin, TNF-α,	↓Pain VAS, ↓Resistin, ⇢⇠TNF-α	NA
	Hosseinzadeh-Attar et al. [127], 2020, Iran	Double-blind RCT	48 obese women with mild–moderate knee OA (*n* = 23, *n* = 25)	1000 mg/day garlic tablet or placebo for 12 weeks	WOMAC * (pain, stiffness, physical function limitation), pain VAS	↓WOMAC * (total, pain, stiffness, physical limitation), ↓pain VAS	NA
Ginger	Altman et al. [129], 2001, USA	Double-blind RCT	261 patients with knee OA (*n* = 130, *n* = 131)	255 mg ginger or placebo 2×/day for 6 weeks	Pain standing VAS, WOMAC * (total, pain, stiffness, physical function limitation), analgesic use	↓pain standing, ↓pain walking, ↓WOMAC * (total, pain stiffness, and physical limitation), ↓analgesic use	↑GI events in ginger group, 59% in ginger group and 37% in placebo group. No serious AEs
	Heidari-Beni et al. [128], 2019, Iran	Double-blind RCT	60 patients with mild–moderate knee OA (*n* = 30, *n* = 30)	Herbal formulation (30 mg curcumin, 7.5 mg gingerols, 3.75 mg piperine) or naproxen (250 mg) 2×/day for 4 weeks	PGE2	↓PGE2 in both groups	NA
Saffron	Poursamimi et al. [123], 2019, Iran	Double-blind RCT	35 patients with primary knee OA (*n* = 18, *n* = 17)	15 mg/day Krocina or placebo for 16 weeks	Pain VAS, CRP, ESR, Treg-cells, Th17-cells, CD8+ T cells, CD3+ CD4+ T cells, GMFI-IL-17, GMFI-FOXP3	↓Pain VAS, ↓CRP, ⇢⇠ESR, ↓Th17%, ↓GMFI-IL-17, ↑Treg-cells, ↑CD4+, ⇢⇠CD8+, ⇢⇠GMFI-FOXP3, ↑Treg/Th17 ratio	NA

Symbols: ↑, increased; ↓, decreased; ⇢⇠, no change. Abbreviations: 30 s chair stand test (CST), 40 m Fast-paced Walking Test (FPWT), Brief Pain Inventory (BPI), C-reactive protein (CRP), erythrocyte sedimentation rate (ESR), Fibromyalgia Impact Questionnaire (FIQ), forkhead box P3 (FoxP3), geometric mean fluorescence intensity (GMFI), Global Fatigue Index (GFI), Hamilton Rating Scale for Depression (HRSD), Health Assessment Questionnaire (HAQ), Hospital Anxiety and Depression Scale (HADS), Interleukin-6 (IL-6), Interleukin -1-beta (IL-Iβ), Japanese Orthopedic Association Score for Osteoarthritic Knees (JOA), Knee Injury Osteoarthritis Outcome Score (KOOS), Malonedialdehyde (MDA), Pain Visual Analogue Scale (VAS), Physical Performance Measurements (PPMs), Prostaglandin E2 (PGE2), reactive oxygen species (ROS), serum glutathione (GSH), superoxide dismutase (SOD), Stand Chair Test (SCT), T helper cells (Th), regulatory T cells (Treg), timed up-and-go test (TUG), tumor necrosis factor alpha (TNF-α), Western Ontario and McMaster Universities Osteoarthritis index (WOMAC). * The severity of arthritis in clinical trials is frequently determined by the Western Ontario and McMaster Universities Osteoarthritis Index (WOMAC), a standardized index for the assessment of the severity of OA symptoms, and the VAS for pain [134].

### 4.3. Fibromyalgia

Depressive symptoms are common in patients with FM. An RCT conducted by Shakiba et al. compared the efficacy of saffron (15 mg 2×/day) versus duloxetine (30 mg 2×/day) in the treatment of patients with FM. There were no significant differences in measurements between the two groups [135]. These results are promising, as saffron showed similar efficacy to serotonin and norepinephrine reuptake inhibitors for the treatment of depression and has an excellent safety profile, as reported by previous studies [136]. In this trial, FM patients experienced comparable improvement in symptoms in both saffron and duloxetine interventions based on the Hamilton Rating Scale for Depression (HRSD), Fibromyalgia Impact Questionnaire (FIQ), Brief Pain Inventory (BPI), Global Fatigue Index (GFI), VAS pain, and Hospital Anxiety and Depression Scale (HADS) depression and HADS anxiety scores [135]. It is important that future studies focus on clarifying the exact mechanisms of action of saffron in the central nervous system in patients with FM and depression in order to provide better evidence for its application in clinical settings. This trial is detailed in Table 3.

Flexofytol, a purified curcumin extract, was investigated in a retrospective study in patients with FM and gout. Among the 62 FM patients treated with Flexofytol (42 mg) four times a day, 66.13% felt that the therapy was beneficial. Fatigue, dizziness, heart palpitations, cramps, and paresthesia in the extremities were improved [137]. This study provides a very limited level of evidence but encourages further research to confirm these results. 

The quality of evidence for the effects of spices and herbs on FM patients is limited due to the poor number of RCTs available and due to the small sample sizes. A 14-year follow-up study comparing the prevalence and patterns of alternative medicine use among patients with FM documents that the use of herbal and dietary supplements has increased dramatically, specifically the use of melatonin, fish oil, garlic, ginger, and flaxseed [138]. For this reason, it is essential that more clinical studies are conducted to evaluate their efficacy and safety in patients with FM.

On this note, following the MD also appears to be effective in reducing FM symptoms [139]. As mentioned above, spices and herbs are a component of the MD, and hence, their effect could also be promising in managing FM symptoms. The effect of *ginger rhizome* has been studied in an experimental mouse model of FM, and this trial provides evidence that the daily consumption of GR complements the anti-nociceptive effect of paracetamol in mice, improves other cognitive disturbances associated with pain, and lowers the inflammatory state generated in an experimental FM model [140]. Likewise, animal studies have demonstrated that curcumin has anti-inflammatory and antioxidant potential in FM-like symptoms by suppressing numerous cell-signaling pathways involved in inflammation [141]. A very limited number of human studies have been conducted to evaluate curcumin’s therapeutic potential in FM patients. Further research is needed to improve the understanding of the disease and the potential effect of spices and herbs on the management of FM.

**Table 3:** Effects of herbs and spices on Fibromyalgia in clinical trials.

Spice	Author, Year, and Country	Study Design	Population, Sample Size (Intervention, Control)	Preparation/Dose (Intervention vs. Control) and Duration	Evaluated Parameters	Main Findings	Reported Adverse Effects (AEs)
Saffron	Shakiba et al. [135], 2018, Iran	Double-Blind RCT	46 patients with FM (*n* = 23, *n* = 23)	15 mg saffron extract or duloxetine 30 mg	HRSD, FIQ, BPI, pain VAS, GFI, HADS anxiety and depression, time x treatment interaction for all parameters.	↓HRSD, ↓FIQ, ↓BPI, ↓pain VAS, ↓GFI, ↓HADS depression, ↓HADS anxiety in both groups. No difference between the groups was observed.	4 in saffron group, 9 in duloxetine group.

Symbol: ↓, decreased. Abbreviations: Brief Pain Inventory (BPI), Fibromyalgia Impact Questionnaire (FIQ), Global Fatigue Index (GFI), Hamilton Rating Scale for Depression (HRSD), Hospital Anxiety and Depression (HAD), Visual Analogue Scale (VAS).

### 4.4. Limitations and Implications for Clinical Practice

Overall, some studies have shown the beneficial effects of herbs and spices in both objective measures (e.g., disease activity, inflammatory biomarkers) and subjective measures (e.g., patients self-reported pain or functional disability). Nevertheless, the available RCTs have a high risk of bias, and more research is required to be able to clearly define the health benefits of herbs and spices. Although the current evidence is exciting, clinicians should interpret these results with caution before translating this knowledge into clinical practice. Conflicting results have been found, and inevitably, there are many unique challenges in clinical nutrition research that must be considered. 

Even though most studies on herbs and spices are conducted through dietary supplements (capsules), many confounding factors still prevail. It is important to note that the nutrients and bioactive compounds found in herbs and spices may be consumed by the study participants through their diet, which could be a confounding factor on results, as the whole diet is not usually controlled in these RCTs. Other confounding factors include the baseline nutritional status of the participants and the history of intake of the nutrient/compound of interest [142]. The assessment of the dietary intake and/or baseline level of the nutrient/compound is of major importance in dietary trials as, contrary to pharmaceutical drug trials in which the drug is absent at baseline, the baseline exposure of nutrients is existent and most likely different across participants [142].

Moreover, as mentioned earlier in this paper, storage and cooking methods impact the bioactive properties of herbs and spices, as well as many other characteristics of the study participants. Dietary and host-related factors, such as habitual dietary pattern, the chemical form of the nutrient, the food matrix, and interactions between nutrients, all impact bioavailability [143] Additionally, while pharmacological drugs have isolated purposes, single targets and are not homeostatically controlled by the body, and nutrients participate in a complex cross-talk that can target all cells and tissues in the body, leading to various effects and outcomes [144] There is no doubt that many challenges arise with the study of dietary compounds and with the selection of the most appropriate study design, as well as its interpretation. To tackle some of these issues, future studies should clearly identify not only the dosage of the herb/spice but describe as much as possible the exact compound that is being used (e.g., if applicable, chemical form, the part of the plant, stage of maturity). Furthermore, baseline characteristics of the study participants should include relevant clinical scores, besides self-reported measures, such as disease activity, if applicable.

Addressing these limitations may lead the way to the implementation of specific recommendations in clinical practice, which cannot yet be truly designed. As of now, clinicians may recommend the culinary use of herbs and spices as a part of an overall healthy dietary pattern, such as the MD; they were shown to be generally safe and are also healthier alternatives to salt, sugar, and fat to enhance food flavor.

## 5. Conclusions

**Figure 2:**
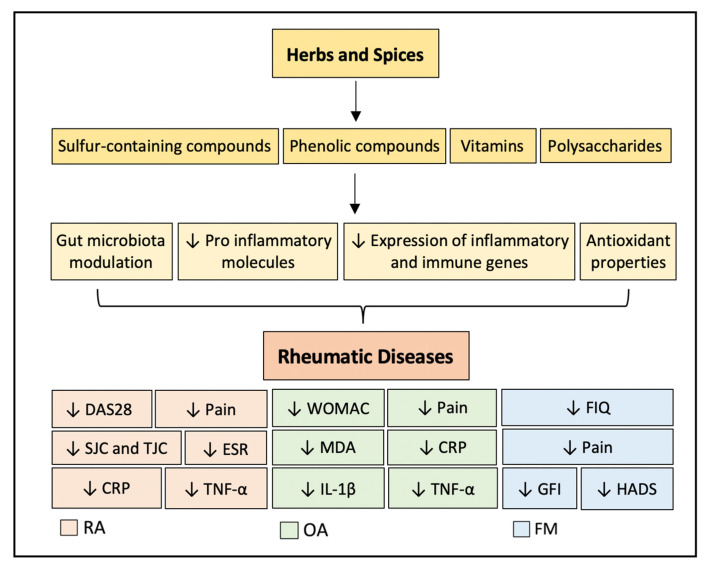
Proposed mechanisms for the influence of herbs and spices on systemic inflammation and their main effects in RA, OA, and FM. Symbol: ↓, decreased. Abbreviations: C-reactive protein (CRP), disease activity score in 28 joints (DAS28), erythrocyte sedimentation rate (ESR), Fibromyalgia (FM), Fibromyalgia Impact Questionnaire (FIQ), Global Fatigue Index (GFI), Hospital Anxiety and Depression Scale (HADS), Interleukin-1 beta (IL-1β), Malondialdehyde (MDA), Osteoarthritis (OA), Rheumatoid arthritis (RA), swollen joint counts (SJCs), tender joint counts (TJCs), tumor necrosis factor alpha (TNF-a).

In this paper, we explore the role of herbs and spices in RDs, as well as the link between these complex disorders, inflammation, and gut microbiota. Figure 2 illustrates some of the possible mechanisms for the influence of herbs and spices on systemic inflammation and their main effects on RDs. Although the results are promising, especially in the context of the many unmet needs of patients with RDs, where achieving and maintaining full remission is rather challenging, the current evidence is insufficient to make recommendations.

Future research should address both supplementation and culinary use of these plants/compounds, discussing relevant topics such as dosage and bioavailability. Future studies should also clearly define baseline disease activity and current pharmacological therapy and clearly specify the culinary herb/spice that is being used. This may contribute to the concrete definition of the most appropriate herb or spice, or mix of both, for RDs. 

## Data Availability

Not applicable.

## Abbreviations

American College of Rheumatology (ACR), Brief Pain Inventory (BPI), C-reactive protein (CRP), *Cinnamic aldehyde (CA)*, cyclooxygenase (COX), disease activity score in 28 joints (DAS28), disease-modifying anti-rheumatic drugs (DMARDs), diallyl sulfide (DAS), diallyl disulfide (DADS), diallyl trisulfide (DATS), docosahexaenoic acid (DHA), eicosapentaenoic acid (EPA), erythrocyte sedimentation rate (ESR), Fibromyalgia syndrome (FM), Fibromyalgia Impact Questionnaire (FIQ), forkhead box P3 (FoxP3), garlic organosulfur compounds (GOSCs), global assessment (GA), Global Fatigue Index (GFI), glutathione (GSH), Hamilton Rating Scale for Depression (HRSD), high-sensitivity C-reactive protein (hs-PCR), homeostatic model assessment for insulin resistance (HOMA-IR), interferon gamma (IFN-γ), Interleukin (IL), Japanese Orthopedic Association Score for Osteoarthritic Knees (JOA), Knee Injury Osteoarthritis Outcome Score (KOOS), lipopolysaccharide (LPS), malondialdehyde (MDA), Mediterranean Diet (MD), natural killer (NK) cells, nuclear factor kappa-light-chain-enhancer of activated B cells (NF-kB), Osteoarthritis (OA), Patient-Reported Outcomes Measurement Information System-29 (PROMIS-29), peroxisome proliferator-activated receptor-gamma (PPAR-γ), polyunsaturated fatty acids (PUFAs), protein kinase C (PKC), Randomized Controlled Trials (RCTs), reactive oxygen species (ROS), regulatory T cells (Tregs), retineic-acid-receptor-related orphan nuclear receptor gamma (RORγt), rheumatic diseases (RDs), Rheumatoid Arthritis (RA), S-allylcysteines (SACs), short-chain fatty acids (SCFAs), superoxide dismutase (SOD), swollen joint counts (SJCs), T helper cells (Th), tender joint counts (TJCs), total antioxidant capacity (TAC), tumor necrosis factor alpha (TNF-a), Visual Analogue Scale (VAS), Western Ontario and McMaster Universities Osteoarthritis index (WOMAC), *γ*-glutamyl-S-allyl-L-cysteines (GSACs).
